# Systemic inflammation and cerebrovascular injury in acute stroke

**DOI:** 10.1590/1806-9282.20260557

**Published:** 2026-08-10

**Authors:** Tuna Demirbas, Neslin Sahin, Fatih Turkmen, Aygul Tantik Pak, Nedim Uzun

**Affiliations:** 1 University of Health Sciences, Istanbul Gaziosmanpasa Training and Research Hospital, Department of Radiology – Istanbul, Türkiye.; 2 University of Health Sciences, Gaziosmanpasa Training and Research Hospital, Department of Neurology – Istanbul, Türkiye.; 3 University of Health Sciences, Gaziosmanpasa Training and Research Hospital, Department of Emergency Medicine – Istanbul, Türkiye.

**Keywords:** Stroke, Cerebral small vessel diseases, Vascular calcification, Inflammation. Biomarkers

## Abstract

**OBJECTIVE:**

Systemic inflammation contributes to cerebrovascular injury, yet cerebral small vessel disease and intracranial arterial calcification are typically evaluated separately. The aim of this study was to investigate the association between systemic inflammatory indices and the combined burden of small vessel disease and intracranial arterial calcification in acute ischemic stroke.

**METHODS:**

This retrospective study included 166 patients with acute anterior circulation ischemic stroke. Total small vessel disease burden (white matter hyperintensities, lacunes, cerebral microbleeds, and enlarged perivascular spaces) was assessed on magnetic resonance imaging. Intracranial arterial calcification severity was evaluated on non-contrast computed tomography using a semiquantitative grading system. Inflammatory indices, including neutrophil-to-lymphocyte ratio and systemic inflammatory response index, were calculated from baseline blood samples. Multivariate regression analyses were performed to identify independent predictors.

**RESULTS:**

A total of 166 patients (mean age 64.5±9.8 years) were included. In multivariate analysis, the neutrophil-to-lymphocyte ratio was an independent predictor of both total small vessel disease burden (β=0.39, p=0.026) and intracranial arterial calcification severity (β=0.181, p=0.030), whereas the systemic inflammatory response index was not significant after adjustment. Additionally, the neutrophil-to-lymphocyte ratio was independently associated with cerebral microbleeds and hypertension, and platelet-related indices were associated with infarct size and enlarged perivascular spaces.

**CONCLUSION:**

Neutrophil-to-lymphocyte ratio is independently associated with both microvascular and macrovascular cerebrovascular injury, supporting an inflammation-driven continuum. It may serve as a practical biomarker for assessing global cerebrovascular disease burden.

## INTRODUCTION

Systemic inflammation is a key link between vascular risk exposure and cerebrovascular injury^1,2^. Pro-inflammatory cytokines promote endothelial dysfunction, oxidative stress, leukocyte adhesion, and blood–brain barrier disruption, contributing to both microvascular degeneration and arterial remodeling^3,4^. These mechanisms are implicated in cerebral small vessel disease (SVD) and intracranial arterial calcification (ICAC)^5,6^. Despite shared pathophysiology, these entities have largely been studied separately.

Previous research on SVD has focused on individual imaging markers or total SVD burden^7^, whereas ICAC studies have emphasized atherosclerotic remodeling and calcification severity^8,9^. Thus, whether these conditions represent interconnected manifestations of a shared inflammatory continuum remains unclear.

This study aimed to evaluate the association between systemic inflammatory indices and the combined burden of SVD and ICAC in acute ischemic stroke, and to determine whether these conditions reflect parallel outcomes of a common inflammatory process rather than distinct vascular entities.

## METHODS

### Study design and population

In this retrospective cross-sectional study, a total of 166 adult patients with acute anterior circulation ischemic stroke admitted to our institution between January 2020 and November 2023 were enrolled. All patients underwent a standardized diagnostic workup, including cranial computed tomography (CT), magnetic resonance imaging (MRI), and routine hematological evaluation upon admission.

To ensure data integrity and minimize confounding, patients with suboptimal image quality, prior neurosurgery, intracranial masses, or cerebral venous thrombosis were excluded. Patients with intracranial hemorrhage outside the ischemic territory were also excluded. Additionally, individuals under 18 years of age or with active infection, autoimmune disease, malignancy, hematological disorders, or immunosuppressive therapy were not included.

### Clinical data collection and risk factor definitions

Demographic and clinical data were collected from electronic medical records at admission, including age, sex, and major vascular risk factors: hypertension (HT), diabetes mellitus (DM), hyperlipidemia, smoking, coronary artery disease (CAD), and atrial fibrillation (AF), all defined according to standard clinical criteria. All variables were verified using electronic records.

### Hematological evaluation and inflammatory indices

Blood samples were obtained within 48 h of imaging at admission. Complete blood count parameters, including neutrophil (N), lymphocyte (L), monocyte (M), and platelet (P) counts, were analyzed using an automated hematology analyzer. Inflammatory indices were calculated as follows: neutrophil-to-lymphocyte ratio (NLR=N/L), lymphocyte-to-monocyte ratio (LMR=L/M), platelet-to-lymphocyte ratio (PLR=P/L), systemic inflammatory response index (SIRI=N×[M/L]), and systemic immune-inflammation index (SIII=N×P/L). All analyses were based on baseline laboratory values, and laboratory assessors were blinded to radiological findings.

### Imaging acquisition and analysis

All patients underwent standardized neuroimaging at admission. Initial evaluation included non-contrast cranial CT and diffusion-weighted imaging (DWI) within 24 h to confirm acute ischemia and exclude hemorrhage, followed by comprehensive MRI within 7 days. CT was performed using 128-slice scanners (GE HealthCare) with 120 kVp and automated tube current modulation. Images were reconstructed at 5 mm for routine evaluation and 1–1.25 mm for detailed assessment of intracranial arterial calcification. MRI was performed on 1.5-T scanners (GE HealthCare) using a standard protocol including T1-weighted, T2-weighted, Fluid-Attenuated Inversion Recovery (FLAIR), DWI (b=0 and 1,000 s/mm²), and Susceptibility-Weighted Imaging (SWI) sequences, with slice thickness of 4–5 mm and 0.5–1 mm interslice gap. All images were independently reviewed by an experienced neuroradiologist blinded to clinical and laboratory data.

### Radiological evaluation

#### Ischemic lesion classification

Ischemic lesions were classified by size and anatomical distribution. Large infarcts were defined as lesions involving major arterial territories or measuring >15 mm. Small infarcts (lacunar infarcts) were defined as lesions ≤15 mm within a single perforating artery territory, according to Standards for Reporting Vascular Changes on Neuroimaging (STRIVE) criteria^10^.

#### Cerebral small vessel disease assessment

Total SVD burden was assessed using a validated scoring system (0–4) based on STRIVE criteria, as described by Staals et al.^11^. One point was assigned for each of the following markers: lacunes, white matter hyperintensities (WMH), enlarged perivascular spaces (EPVS), and cerebral microbleeds (CMB). Lacunes were defined as 3–15 mm subcortical fluid-filled cavities with cerebrospinal fluid signal. WMH were evaluated on FLAIR images and considered significant if the Fazekas score ≥2. EPVS were assessed in the basal ganglia, with ≥11 lesions considered moderate-to-severe. CMBs were identified on SWI as small (<10 mm) hypointense lesions not related to vascular flow voids or calcifications.

#### Intracranial arterial calcification

ICAC was evaluated on non-contrast thin-section CT at the carotid siphon using the modified Woodcock grading system^12^. Calcifications were graded as 0 (absent), 1 (mild; <25%), 2 (moderate; 25–50%), and 3 (severe; >50% or protrusion). For analysis, grade 3 was defined as high burden and grades 0–2 as low-to-moderate. Images were reviewed by a blinded neuroradiologist.

### Sample size considerations

An a priori sample size calculation indicated that at least 84 participants were required (effect size r=0.30, α=0.05, power=80%). A total of 166 patients were included, exceeding this requirement and providing adequate statistical power. The sample size was sufficient for multivariate regression analyses, ensuring reliable estimation of independent predictors of total SVD score and ICAC severity.

### Statistical analysis

Statistical analyses were performed using Statistical Package for the Social Sciences version 26.0 (IBM Corp., Armonk, NY, USA). Normality was assessed using the Kolmogorov-Smirnov test and histogram inspection. Continuous variables were expressed as mean±standard deviation or median (range), and categorical variables as frequencies and percentages.

Group comparisons were performed using the independent-samples t-test or Mann-Whitney U test, and analysis of variance or Kruskal-Wallis test for multiple groups. Categorical variables were analyzed using the chi-square or Fisher’s exact test.

Multivariate linear regression was used to identify independent predictors of continuous outcomes (total SVD score and ICAC severity), while logistic regression was applied for dichotomous outcomes (SVD markers and vascular risk factors). Results were reported as odds ratios with 95%CI.

Variables with p<0.10 in univariate analysis were included in multivariate models. Multicollinearity was assessed using the variance inflation factor, and p<0.05 was considered statistically significant.

### Ethics approval and consent to participate

The study was conducted in accordance with the Declaration of Helsinki and approved by the Institutional Review Board (Approval No: 29; February 5, 2025). The requirement for informed consent was waived due to the retrospective design and use of anonymized data.

## RESULTS

### Baseline demographic, clinical, laboratory, and radiological characteristics

A total of 166 patients (56.6% male) with a mean age of 64.5±9.8 years were included. There was no significant age difference between males and females (p=0.185), and 62% of patients were aged over 60 years.

HT (77.7%) was the most prevalent comorbidity, followed by hyperlipidemia (64.5%) and DM (42.2%). Smoking, CAD, and AF were present in 36.1, 30.1, and 25.3% of patients, respectively.

Mean laboratory values were as follows: White blood cell count 8.36±2.56×10⁹/L, neutrophils 5.43±2.32×10⁹/L, lymphocytes 2.17±0.78×10⁹/L, monocytes 0.54±0.19×10⁹/L, and platelets 231.97±71.92×10⁹/L. Inflammatory indices included NLR 2.87±1.90, LMR 4.38±1.89, PLR 120.35±62.17, SIRI 1.61±1.40, and SIII 663.14±474.16.

Neuroimaging showed that 83.1% of infarcts were large. The prevalence of SVD markers was: lacunes 36.7%, EPVS 45.2%, WMH 44.6%, and CMB 13.3%. Mild and severe ICAC were observed in 53.6 and 36.1% of patients, respectively. The intimal pattern was more frequent than the medial pattern, without statistical significance (p=0.07).

### Association between clinical factors and inflammatory indices

Systemic inflammatory indices were significantly associated with several clinical parameters. Patients with HT showed higher NLR (p<0.001), PLR (p=0.042), SIRI (p=0.018), and SIII (p=0.005), whereas LMR was higher in non-hypertensive individuals (p=0.046). SIRI was also elevated in patients with AF (p=0.017). No significant associations were observed for diabetes, hyperlipidemia, smoking, or CAD (all p>0.05).

### Radiological findings and multivariate analyses

Patients with large infarcts showed significantly higher PLR (p<0.001) and SIII (p=0.008). WMH was associated with higher NLR (p=0.043) and lower LMR (p=0.044), whereas no significant associations were found for lacunes, EPVS, or CMB in univariate analyses (all p>0.05). Similarly, inflammatory indices were not associated with ICAC severity or pattern in univariate analyses.

In multivariate models adjusted for age, sex, and vascular risk factors, NLR emerged as an independent predictor of both total SVD burden (β=0.39, p=0.026, 95%CI 0.046–0.734) and ICAC severity (β=0.181, p=0.030, 95%CI 0.018–0.344). SIRI showed a non-significant trend for ICAC (p=0.059) (Table 1).

**Table 1:** Multivariate regression analysis of inflammatory indices and radiological parameters.

Outcome variable: total SVD score						
Predictor	B	SE	β	t	p	95%CI
NLR	0.390	0.174	0.556	2.240	0.026^*^	0.046–0.734
LMR	0.041	0.082	0.058	0.500	0.618	-0.121–0.203
PLR	0.005	0.003	0.236	1.489	0.139	-0.002–0.012
SIRI	-0.100	0.183	-0.106	-0.549	0.584	-0.461–0.260
SIII	-0.002	0.001	-0.538	-1.864	0.064	-0.003–0.000
Outcome variable: ICAC severity						
Predictor	B	SE	Β	t	p	95%CI
NLR	0.181	0.082	0.544	2.195	0.030^*^	0.018–0.344
LMR	-0.041	0.039	-0.122	-1.051	0.295	-0.118–0.036
PLR	-0.001	0.002	-0.051	-0.321	0.748	-0.004–0.003
SIRI	-0.165	0.087	-0.366	-1.905	0.059	-0.336–0.006
SIII	0.000	0.000	-0.172	-0.596	0.552	-0.001–0.001
Outcome variable: ICAC score						
Predictor	B	SE	Β	t	p	95%CI
NLR	0.068	0.084	0.199	0.802	0.424	-0.099–0.234
LMR	-0.078	0.04	-0.229	-1.965	0.051	-0.157–0.000
PLR	-0.002	0.002	-0.177	-1.114	0.267	-0.005–0.001
SIRI	-0.133	0.089	-0.29	-1.504	0.134	-0.308–0.042
SIII	0.000	0.000	0.175	0.608	0.544	-0.001–0.001

*Statistically significant (p<0.05). NLR: neutrophil-to-lymphocyte ratio; LMR: lymphocyte-to-monocyte ratio; PLR: platelet-to-lymphocyte ratio; SIRI: systemic inflammatory response index; SIII: systemic immune-inflammation index; SVD: small vessel disease; ICAC: intracranial arterial calcification; SE: standard error; CI: confidence interval.

Logistic regression analysis demonstrated that NLR (OR 2.476, p=0.003), PLR (OR 1.012, p=0.046), and SIII (OR 0.997, p=0.015) were independently associated with EPVS. Additionally, NLR was an independent predictor of CMB (OR 2.521, p=0.037) and HT (OR 3.174, p=0.012).

## DISCUSSION

This study demonstrates a shared inflammatory mechanism underlying both microvascular and macrovascular cerebrovascular injury. NLR emerged as an independent predictor of both total SVD burden and ICAC severity after adjustment for vascular risk factors.

Previous studies have generally evaluated SVD and ICAC separately^13,14^. Our findings suggest that these conditions may represent interconnected manifestations of a shared inflammatory continuum^15^. Systemic inflammation contributes to endothelial dysfunction, oxidative stress, and blood–brain barrier disruption, promoting SVD, while also driving vascular remodeling and arterial calcification^16,17^.

NLR reflects the balance between innate and adaptive immunity and showed the strongest associations across both domains. Supporting this, Liu et al.^18^ reported that higher NLR is associated with poorer outcomes, whereas Chen et al.^19^ showed that lower NLR predicts favorable recovery. These findings suggest that NLR reflects overall cerebrovascular disease burden.

A key finding was the discrepancy between univariate and multivariate analyses for ICAC. While no association was observed in univariate analysis, NLR became significant after adjustment, indicating that inflammatory effects may be masked by traditional risk factors^20^.

WMH was associated with higher NLR and lower LMR, consistent with prior reports linking inflammation to white matter injury^21^. The association between NLR and CMB further suggests a role of inflammation in blood–brain barrier disruption.

Inflammatory indices were also associated with infarct size. Elevated PLR and SIII in large infarcts indicate a pro-inflammatory and pro-thrombotic state. However, inconsistent findings regarding PLR have been reported^22^, suggesting it may reflect acute rather than long-term processes.

Clinically, NLR may serve as a simple and practical biomarker for identifying patients with higher global cerebrovascular burden and potentially worse prognosis.

## CONCLUSION

Systemic inflammation, reflected by NLR, is independently associated with both cerebral small vessel disease burden and intracranial arterial calcification severity in acute ischemic stroke, supporting an inflammation-driven continuum of cerebrovascular injury. As a simple and accessible biomarker, NLR may reflect global cerebrovascular burden and aid in risk stratification. Further prospective studies are needed to confirm these findings and explore potential therapeutic implications.

### Limitations

Limitations include the retrospective design, single-center setting, lack of longitudinal data, potential residual confounding, single-observer assessment, and use of visual grading methods.

## Data Availability

The datasets generated and/or analyzed during the current study are available from the corresponding author upon reasonable request.
